# 1-(4-Bromo-3-chloro­phen­yl)-3-meth­oxy-3-methyl­urea (chlorbromuron)

**DOI:** 10.1107/S1600536810030606

**Published:** 2010-08-11

**Authors:** Harrison M. Black, Russell G. Baughman

**Affiliations:** a Department of Chemistry, Truman State University, Kirksville, MO 63501-4221, USA

## Abstract

In the title urea-based herbicide, C_9_H_10_BrClN_2_O_2_, there exist multiple inter- and intra­molecular inter­actions. Most notably, the intra­molecular hydrogen bond between the urea carbonyl O atom and an aromatic H atom affects the planarity and torsion angles of the mol­ecule by restricting rotations about the Ar—secondary amine N and the secondary amine N and the carbonyl C. The two N atoms in the urea fragment are in different environments. One is planar; the other, pseudo-*C*_3v_. It is likely that the different nitro­gen-atom geometries and the restricted rotations within the mol­ecule impact the bioactivity of chlorbromuron.

## Related literature

The structure of the title compound, chlorbromuron, was determined as part of a larger project on the crystal and mol­ecular structures of a series of herbicides, see: Baughman & Yu (1988 ▶ and references cited therein). Chlorbromuron is a urea-based herbicide that acts to inhibit photosynthesis and the oxidation of water to oxygen during the Hill reaction, see: Metcalf (1971 ▶). Typically one or more hydrogen bonds form between the –NH– or C=O groups in the urea-based herbicides and an active site in the protein, see: Good (1961 ▶).
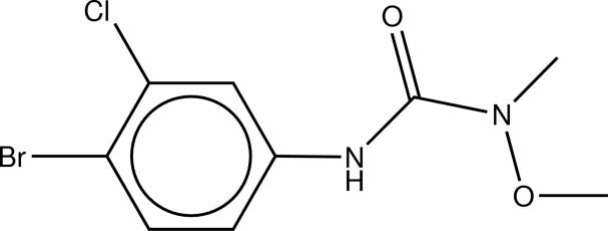


## Experimental

### 

#### Crystal data

C_9_H_10_BrClN_2_O_2_
*M* *_r_* = 293.55
Orthorhombic, 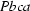

*a* = 11.3872 (7) Å
*b* = 9.5037 (5) Å
*c* = 21.512 (2) Å
*V* = 2328.0 (3) Å^3^
*Z* = 8
Mo *K*α radiation
μ = 3.74 mm^−1^
*T* = 295 K
0.44 × 0.44 × 0.42 mm

#### Data collection

Bruker P4 diffractometer
Absorption correction: integration (*XSHELL*; Bruker, 1999 ▶) *T*_min_ = 0.224, *T*_max_ = 0.331
2643 measured reflections
2017 independent reflections
1230 reflections with *I* > 2σ(*I*)
*R*_int_ = 0.107
3 standard reflections every 100 reflections  intensity decay: 1.3%

#### Refinement

*R*[*F* ^2^ > 2σ(*F* ^2^)] = 0.065
*wR*(*F* ^2^) = 0.173
*S* = 0.99
2017 reflections
136 parameters
H-atom parameters constrained
Δρ_max_ = 1.13 e Å^−3^
Δρ_min_ = −0.63 e Å^−3^

### 

Data collection: *XSCANS* (Bruker, 1996 ▶); cell refinement: *XSCANS*; data reduction: *XSCANS*; program(s) used to solve structure: *SHELXS86* (Sheldrick, 2008 ▶); program(s) used to refine structure: *SHELXL97* (Sheldrick, 2008 ▶); molecular graphics: *SHELXTL/PC* (Sheldrick, 2008 ▶); software used to prepare material for publication: *SHELXTL/PC* and *SHELXL97*.

## Supplementary Material

Crystal structure: contains datablocks I, global. DOI: 10.1107/S1600536810030606/bv2148sup1.cif

Structure factors: contains datablocks I. DOI: 10.1107/S1600536810030606/bv2148Isup2.hkl

Additional supplementary materials:  crystallographic information; 3D view; checkCIF report

## Figures and Tables

**Table 1:** Selected torsion angles (°)

C9—O2—N2—C7	−124.6 (6)
C9—O2—N2—C8	97.8 (7)
C7—N1—C1—C2	23.3 (9)
O2—N2—C7—N1	20.2 (7)

**Table 2:** Hydrogen-bond geometry (Å, °)

*D*—H⋯*A*	*D*—H	H⋯*A*	*D*⋯*A*	*D*—H⋯*A*
N1—H1⋯O1^i^	0.86	2.11	2.902 (7)	153
N1—H1⋯O2	0.86	2.18	2.573 (6)	108
C2—H2⋯O1	0.93	2.35	2.873 (7)	115

Symmetry code: (i) 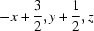.

**Fig. 1.**
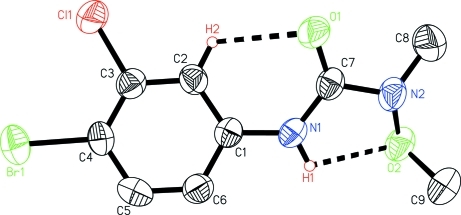
The asymmetric unit of (I). Displacement ellipsoids are drawn at the 50% probability levels; H atoms are drawn as small spheres of arbitrary radius.

## supplementary crystallographic information

### Comment

The crystal structure of 3-(4-bromo-3-chlorophenyl)-1-methoxy-1- methylurea, chlorbromuron (I), was determined as part of a larger project (Baughman and Yu, 1988 and references cited therein) that has focused on the crystal and molecular structures of a series of herbicides. Chlorbromuron is a urea-based herbicide that acts to inhibit photosynthesis and the oxidation of water to oxygen during the Hill reaction (Metcalf, 1971). Typically one or more hydrogen bonds form between the –NH– or C=O groups in the urea-based herbicides and an active site in the protein (Good, 1961).

Molecules in the unit cell utilize intermolecular hydrogen bonds, while the molecule contains intramolecular hydrogen bonds. The O1···H1 intermolecular hydrogen bond and the O2···H1 and O1···H2 intramolecular hydrogen bonds affect the torsion angles (Table 1). The O1···H2 hydrogen bond is interesting because it likely causes H2/C2/C1/N1/C7/O1 to be nearly planar (r.m.s. deviation = 0.167 Å). Similarly, the O2—N2—C7—N1 torsion angle of 20.2 (7)° and near planarity of H1A/N1/C7/N2/O2 (r.m.s. = 0.100 Å) indicate the effect of the O2···H1 hydrogen bond.

Since the O1···H2 and O2···H1 intramolecular hydrogen bonds limit rotation, the structure of (I) presented here may also be close to that *in vitro* and/or *in vivo*. The limiting of the rotational degrees of freedom about the C1—N1, N1—C7, and C7—N2 bonds may influence the bioactivity of (I). A very weak intermolecular interaction between Cl1 and H9A likely exists, but, at a distance of 3.15 Å, is a little too long to be considered a true hydrogen bond, but may have some impact on packing.

Structurally significant angles around N2 indicate a pseudo-C_3v_ shape (Fig. 1, Table 1). However, the geometry around N1 is planar. Although the C4—C3—Cl1 angle [121.1 (5)°] is ~2σ greater than the ideal angle of 120°, the C3—C4—Br1 angle [122.3 (5)°] is ~5σ greater than ideal angle, which indicates some steric and charge repulsion in this portion of the molecule.

### Experimental

Crystals were grown by slow evaporation of a solution in EtOH.

### Refinement

Approximate positions of all H's were first obtained from a difference map, then placed into "ideal" positions. Bond lengths were constrained at 0.93 Å (AFIX 43) for aromatic C—H's, at 0.96 Å (AFIX 137) for methyl C—H's, and 0.86 Å (AFIX 43) for the N—H.

*U*_iso_(H) were fixed at 1.5*U*_eq_(parent) for OH and methyl H's, and 1.2 *U*_eq_(parent) for all other H's.

In the final stages of refinement for (I), 14 very small or negative F_o_'s were deemed to be in high disagreement and were eliminated from final refinement.

Percent decay of the three standards was calculated as the average of their σ(*I*)'s.

### Crystal data

**Table:**

C_9_H_10_BrClN_2_O_2_	*F*(000) = 1168
*M**_r_* = 293.55	*D*_x_ = 1.675 Mg m^−^^3^
Orthorhombic, *P**b**c**a*	Mo *K*α radiation, λ = 0.71073 Å
Hall symbol: -P 2ac 2ab	Cell parameters from 100 reflections
*a* = 11.3872 (7) Å	θ = 10.0–15.8°
*b* = 9.5037 (5) Å	µ = 3.74 mm^−^^1^
*c* = 21.512 (2) Å	*T* = 295 K
*V* = 2328.0 (3) Å^3^	Rectangular prism, colorless
*Z* = 8	0.44 × 0.44 × 0.42 mm

### Data collection

**Table:**

Bruker P4 diffractometer	1230 reflections with *I* > 2σ(*I*)
Radiation source: normal-focus sealed tube	*R*_int_ = 0.107
graphite	θ_max_ = 25.0°, θ_min_ = 1.9°
Ω scans	*h* = −1→13
Absorption correction: integration (XSHELL; Bruker, 1999)	*k* = −11→1
*T*_min_ = 0.224, *T*_max_ = 0.331	*l* = −25→1
2643 measured reflections	3 standard reflections every 100 reflections
2017 independent reflections	intensity decay: 1.3%

### Refinement

**Table:**

Refinement on *F*^2^	Primary atom site location: structure-invariant direct methods
Least-squares matrix: full	Secondary atom site location: difference Fourier map
*R*[*F*^2^ > 2σ(*F*^2^)] = 0.065	Hydrogen site location: inferred from neighbouring sites
*wR*(*F*^2^) = 0.173	H-atom parameters constrained
*S* = 0.99	*w* = 1/[σ^2^(*F*_o_^2^) + (0.1011*P*)^2^ + 0.010*P*] where *P* = (*F*_o_^2^ + 2*F*_c_^2^)/3
2017 reflections	(Δ/σ)_max_ < 0.001
136 parameters	Δρ_max_ = 1.13 e Å^−^^3^
0 restraints	Δρ_min_ = −0.63 e Å^−^^3^

### Special details

**Table:**

Geometry. All s.u.'s (except the s.u. in the dihedral angle between two l.s. planes) are estimated using the full covariance matrix. The cell s.u.'s are taken into account individually in the estimation of s.u.'s in distances, angles and torsion angles; correlations between s.u.'s in cell parameters are only used when they are defined by crystal symmetry. An approximate (isotropic) treatment of cell s.u.'s is used for estimating s.u.'s involving l.s. planes.
Refinement. Refinement of *F*^2^ against ALL reflections. The weighted *R*-factor *wR* and goodness of fit, *S*, are based on *F*^2^, conventional *R*-factors *R* are based on *F*, with *F* set to zero for negative *F*^2^. The threshold expression of *F*^2^ > 2σ(*F*^2^) is used only for calculating *R*-factors(gt) *etc*. and is not relevant to the choice of reflections for refinement. *R*-factors based on *F*^2^ are statistically about twice as large as those based on *F*, and *R*-factors based on ALL data will be even larger.

### Fractional atomic coordinates and isotropic or equivalent isotropic displacement parameters (Å^2^)

**Table:**

	*x*	*y*	*z*	*U*_iso_*/*U*_eq_	
Br1	0.99321 (6)	0.13975 (8)	0.40066 (3)	0.0648 (4)	
Cl1	0.75978 (19)	−0.0374 (2)	0.44369 (7)	0.0690 (6)	
O1	0.6655 (4)	0.0148 (4)	0.6675 (2)	0.0571 (12)	
O2	0.6463 (4)	0.3363 (4)	0.7430 (2)	0.0560 (12)	
N1	0.7597 (4)	0.2249 (5)	0.6520 (2)	0.0424 (12)	
H1	0.7741	0.3050	0.6690	0.051*	
N2	0.6419 (5)	0.1877 (5)	0.7384 (2)	0.0501 (14)	
C1	0.8124 (5)	0.1972 (6)	0.5945 (3)	0.0389 (14)	
C2	0.7677 (6)	0.1002 (6)	0.5513 (3)	0.0449 (15)	
H2	0.7011	0.0479	0.5609	0.054*	
C3	0.8216 (5)	0.0839 (6)	0.4948 (3)	0.0457 (15)	
C4	0.9200 (5)	0.1589 (6)	0.4788 (3)	0.0443 (15)	
C5	0.9659 (5)	0.2525 (7)	0.5221 (3)	0.0506 (17)	
H5	1.0328	0.3040	0.5121	0.061*	
C6	0.9133 (6)	0.2707 (6)	0.5784 (3)	0.0466 (15)	
H6	0.9457	0.3331	0.6070	0.056*	
C7	0.6870 (6)	0.1363 (7)	0.6830 (3)	0.0429 (15)	
C8	0.5314 (7)	0.1313 (8)	0.7610 (4)	0.074 (2)	
H8A	0.5320	0.0306	0.7571	0.111*	
H8B	0.5204	0.1565	0.8038	0.111*	
H8C	0.4684	0.1693	0.7366	0.111*	
C9	0.7083 (8)	0.3723 (8)	0.7985 (3)	0.079 (2)	
H9A	0.7121	0.4729	0.8023	0.118*	
H9B	0.6691	0.3336	0.8341	0.118*	
H9C	0.7864	0.3348	0.7960	0.118*	

### Atomic displacement parameters (Å^2^)

**Table:**

	*U*^11^	*U*^22^	*U*^33^	*U*^12^	*U*^13^	*U*^23^
Br1	0.0657 (5)	0.0802 (6)	0.0485 (5)	0.0019 (4)	0.0147 (4)	0.0053 (4)
Cl1	0.0940 (14)	0.0718 (12)	0.0412 (9)	−0.0225 (10)	0.0052 (10)	−0.0125 (9)
O1	0.084 (3)	0.038 (3)	0.049 (3)	−0.006 (2)	0.007 (3)	−0.005 (2)
O2	0.071 (3)	0.047 (2)	0.051 (3)	0.007 (2)	0.003 (2)	−0.013 (2)
N1	0.049 (3)	0.037 (3)	0.041 (3)	0.003 (2)	−0.001 (3)	−0.008 (2)
N2	0.065 (4)	0.042 (3)	0.043 (3)	0.006 (3)	0.009 (3)	−0.007 (2)
C1	0.043 (3)	0.033 (3)	0.041 (3)	0.008 (3)	−0.001 (3)	0.000 (3)
C2	0.051 (4)	0.042 (3)	0.042 (3)	−0.002 (3)	0.005 (3)	0.005 (3)
C3	0.053 (4)	0.043 (3)	0.041 (4)	0.000 (3)	−0.002 (3)	0.001 (3)
C4	0.041 (4)	0.051 (4)	0.040 (3)	0.009 (3)	0.005 (3)	0.002 (3)
C5	0.039 (3)	0.047 (4)	0.065 (5)	−0.006 (3)	0.000 (3)	0.009 (3)
C6	0.050 (4)	0.046 (4)	0.045 (4)	−0.003 (3)	−0.003 (3)	−0.002 (3)
C7	0.052 (4)	0.042 (4)	0.035 (3)	0.007 (3)	−0.004 (3)	0.000 (3)
C8	0.077 (5)	0.074 (5)	0.070 (5)	−0.009 (4)	0.024 (4)	−0.009 (4)
C9	0.094 (6)	0.088 (6)	0.054 (5)	−0.018 (5)	0.003 (4)	−0.023 (4)

### Geometric parameters (Å, °)

**Table:**

Br1—C4	1.885 (6)	C2—H2	0.9303
Cl1—C3	1.741 (6)	C3—C4	1.372 (9)
O1—C7	1.227 (7)	C4—C5	1.390 (9)
O2—N2	1.417 (6)	C5—C6	1.363 (9)
O2—C9	1.428 (8)	C5—H5	0.9302
N1—C7	1.356 (8)	C6—H6	0.9302
N1—C1	1.401 (7)	C8—H8A	0.9604
N1—H1	0.8602	C8—H8B	0.9599
N2—C7	1.385 (7)	C8—H8C	0.9601
N2—C8	1.452 (9)	C9—H9A	0.9605
C1—C6	1.388 (8)	C9—H9B	0.9600
C1—C2	1.404 (8)	C9—H9C	0.9602
C2—C3	1.372 (8)		
			
N2—O2—C9	108.4 (5)	C6—C5—H5	119.9
C7—N1—C1	125.4 (5)	C4—C5—H5	119.3
C7—N1—H1	117.2	C5—C6—C1	121.4 (6)
C1—N1—H1	117.4	C5—C6—H6	119.6
C7—N2—O2	113.5 (5)	C1—C6—H6	119.0
C7—N2—C8	118.6 (6)	O1—C7—N1	124.9 (5)
O2—N2—C8	112.0 (5)	O1—C7—N2	119.5 (6)
C6—C1—N1	118.7 (5)	N1—C7—N2	115.4 (5)
C6—C1—C2	117.8 (6)	N2—C8—H8A	109.4
N1—C1—C2	123.5 (5)	N2—C8—H8B	110.0
C3—C2—C1	119.9 (6)	H8A—C8—H8B	109.5
C3—C2—H2	120.1	N2—C8—H8C	109.0
C1—C2—H2	120.0	H8A—C8—H8C	109.5
C4—C3—C2	121.9 (6)	H8B—C8—H8C	109.5
C4—C3—Cl1	121.1 (5)	O2—C9—H9A	109.4
C2—C3—Cl1	117.0 (5)	O2—C9—H9B	110.2
C3—C4—C5	118.1 (6)	H9A—C9—H9B	109.5
C3—C4—Br1	122.3 (5)	O2—C9—H9C	108.8
C5—C4—Br1	119.6 (5)	H9A—C9—H9C	109.5
C6—C5—C4	120.8 (6)	H9B—C9—H9C	109.5
			
C9—O2—N2—C7	−124.6 (6)	C3—C4—C5—C6	−1.1 (9)
C9—O2—N2—C8	97.8 (7)	Br1—C4—C5—C6	178.5 (5)
C7—N1—C1—C6	−157.8 (6)	C4—C5—C6—C1	−0.1 (9)
C7—N1—C1—C2	23.3 (9)	N1—C1—C6—C5	−177.4 (5)
C6—C1—C2—C3	−1.8 (9)	C2—C1—C6—C5	1.6 (9)
N1—C1—C2—C3	177.0 (5)	C1—N1—C7—O1	6.7 (10)
C1—C2—C3—C4	0.7 (9)	C1—N1—C7—N2	−178.0 (5)
C1—C2—C3—Cl1	−179.4 (5)	O2—N2—C7—O1	−164.2 (5)
C2—C3—C4—C5	0.8 (9)	C8—N2—C7—O1	−29.6 (9)
Cl1—C3—C4—C5	−179.1 (5)	O2—N2—C7—N1	20.2 (7)
C2—C3—C4—Br1	−178.8 (5)	C8—N2—C7—N1	154.8 (6)
Cl1—C3—C4—Br1	1.3 (8)		

### Hydrogen-bond geometry (Å, °)

**Table:**

*D*—H···*A*	*D*—H	H···*A*	*D*···*A*	*D*—H···*A*
N1—H1···O1^i^	0.86	2.11	2.902 (7)	153
N1—H1···O2	0.86	2.18	2.573 (6)	108
C2—H2···O1	0.93	2.35	2.873 (7)	115

Symmetry codes: (i) −*x*+3/2, *y*+1/2, *z*.
